# Seasonal environmental transitions and metabolic plasticity in a sea-ice alga from an individual cell perspective

**DOI:** 10.1038/s41598-024-65273-0

**Published:** 2024-07-01

**Authors:** Rebecca J. Duncan, Janne E. Søreide, Daniel A. Nielsen, Øystein Varpe, Józef Wiktor, Mark J. Tobin, Vanessa Pitusi, Katherina Petrou

**Affiliations:** 1 School of Life Sciences, University of Technology Sydney, Building 7, 67 Thomas St, Ultimo, NSW 2007 Australia; 2 Department of Arctic Biology, The University Centre in Svalbard, Longyearbyen, Norway; 3 Department of Biological Sciences, University of Bergen, Bergen, Norway; 4 Norwegian Institute for Nature Research, Bergen, Norway; 5 Institute of Oceanology, Polish Academy of Sciences, Sopot, Poland; 6 Department of Arctic and Marine Biology, University in Tromsø, Tromsø, Norway; 7 ANSTO-Australian Synchrotron, Clayton, VIC Australia

**Keywords:** Microalgae, Svalbard, *Nitzschia frigida*, Biogeochemical cycling, Sea ice, Arctic, Ecophysiology, Microbial ecology

## Abstract

Sea-ice microalgae are a key source of energy and nutrient supply to polar marine food webs, particularly during spring, prior to open-water phytoplankton blooms. The nutritional quality of microalgae as a food source depends on their biomolecular (lipid:protein:carbohydrate) composition. In this study, we used synchrotron-based Fourier transform infra-red microspectroscopy (s-FTIR) to measure the biomolecular content of a dominant sea-ice taxa, *Nitzschia frigida,* from natural land-fast ice communities throughout the Arctic spring season. Repeated sampling over six weeks from an inner (relatively stable) and an outer (relatively dynamic) fjord site revealed high intra-specific variability in biomolecular content, elucidating the plasticity of *N. frigida* to adjust to the dynamic sea ice and water conditions. Environmental triggers indicating the end of productivity in the ice and onset of ice melt, including nitrogen limitation and increased water temperature, drove an increase in lipid and fatty acids stores, and a decline in protein and carbohydrate content. In the context of climate change and the predicted Atlantification of the Arctic, dynamic mixing and abrupt warmer water advection could truncate these important end-of-season environmental shifts, causing the algae to be released from the ice prior to adequate lipid storage, influencing carbon transfer through the polar marine system.

## Introduction

The last half century has seen ocean temperatures rise and circulation patterns shift, resulting in a rapid decline in Arctic sea ice extent, thickness and area^1–3^. The increasing temperatures and advection of warmer, more saline Atlantic waters (AW) into the Arctic means that sea ice has become more prone to summer melting^4^, which has resulted in a decline in multi-year sea ice (MYI; sea ice which has survived multiple summers)^5^. Since 1999, the extent of MYI has diminished by ~ 50%^2,6^, and now covers less than a third of the Arctic Ocean (AO)^7^, meaning that first year ice (FYI; sea ice which completely melts each summer), which is thinner and more sensitive to changes in the physical environment, now dominates the Arctic^2,3,8^. Warmer surface water temperatures impede Arctic sea ice formation delaying freezing while also causing earlier melting^3^. This contracted period of FYI, with shortened timeframes between ice formation (late winter), substantial daylight returning to the Arctic (early spring) and melting of the ice from below (late spring/summer)^9^, results in a reduced window of productivity for sea ice associated ecosystems. These warming-associated changes in Arctic sea ice conditions have implications for the seasonal productivity and ecology of the polar marine ecosystem^10,11^.

Sea ice forms an important habitat for microalgae, which represent the primary source of energy for the marine ecosystem in the early spring, in the absence of other sources of primary production and prior to the late-spring/summer open-water phytoplankton blooms^9,12^. Through prolonging polar marine biological production^13,14^ and this provision of food during the early phases of seasonal zooplankton reproduction, sea ice microalgae are an important factor in the quality and quantity of secondary production^15–17^. The nutritional quality of microalgae as a food source is dependent on which species are present and their individual elemental stoichiometry, which reflects their biomolecular (lipid:protein:carbohydrate) composition^18^. Cell stoichiometry, and thus biomolecular composition, is sensitive to changes in environment, such as light^19^, temperature^20,21^ and nutrient availability^22,23^, with shifts in each parameter affecting biomolecular stores in different ways. In sea ice microalgae, increasing irradiance transmitted through the ice can increase lipid production^19^, nutrient limitation has been shown to decrease protein and increase fatty acid (FA) content^22,24^, whilst increasing ocean temperatures have been observed to increase protein content and decrease FA content^20,21,25^. As such, shifts in the biomolecular composition of sea ice microalgae as a result of changing sea ice-surface ocean conditions, invariably influences Arctic food webs, local biogeochemistry and plays a vital role in determining the productivity of polar marine ecosystems (^26^ and references therein).

In natural systems, microalgal communities form complex functional networks, making it challenging to obtain knowledge on species’ phenomes and metabolomes in a dynamic and multivariate environment. Species and communities adapt to changing environmental conditions either through phenotypic plasticity or genetically^27^. Seasonally, it is phenotypic adjustments to changes in environment that dominate, but the capacity for such plasticity varies across and within species. Therefore, to gain a systematic understanding of metabolic plasticity and uncover the phenome (set of traits modifiable for enhanced growth and survival) in natural biological systems, we need single-celled multiparameter measurements that can probe the changes occurring in individual cells in response to environmental shifts.

Here we use synchrotron-based Fourier transform infra-red microspectroscopy to measure the biomolecular content of *Nitzschia frigida* from natural sea ice habitat over the Arctic spring. *N. frigida* is one of the most prolific and ubiquitous Arctic sea ice diatoms, typically dominating first year sea ice assemblages^9,28–30^. It is a colony-forming pennate diatom which can often be observed as single cells early in the spring, increasingly creating arborescent colonies as spring progresses. In contrast to taxa such as *Chaetoceros* spp., *Thalassiosira* spp. and dinoflagellates that create resting spores to survive the long winter darkness^31,32^, *N. frigida* are thought to survive the winter in the epibenthic region, primarily through storing energy and reducing metabolic rate^31,33^. This energy storage strategy means that in addition to contributing substantially to spring primary production in the ice, following their release during ice melt, they strongly contribute to carbon flux as they are either consumed during descent^34^ or exported to the benthos^35,36^. In this study, we investigated changes in the biomolecular content of *N. frigida* throughout the spring sea ice season until ice-melt (March–May), tracing the seasonal progression of nutritional content to document potential shifts in energy allocation that may link to survival during polar darkness.

## Materials & methods

### Study area

**Figure 1:**
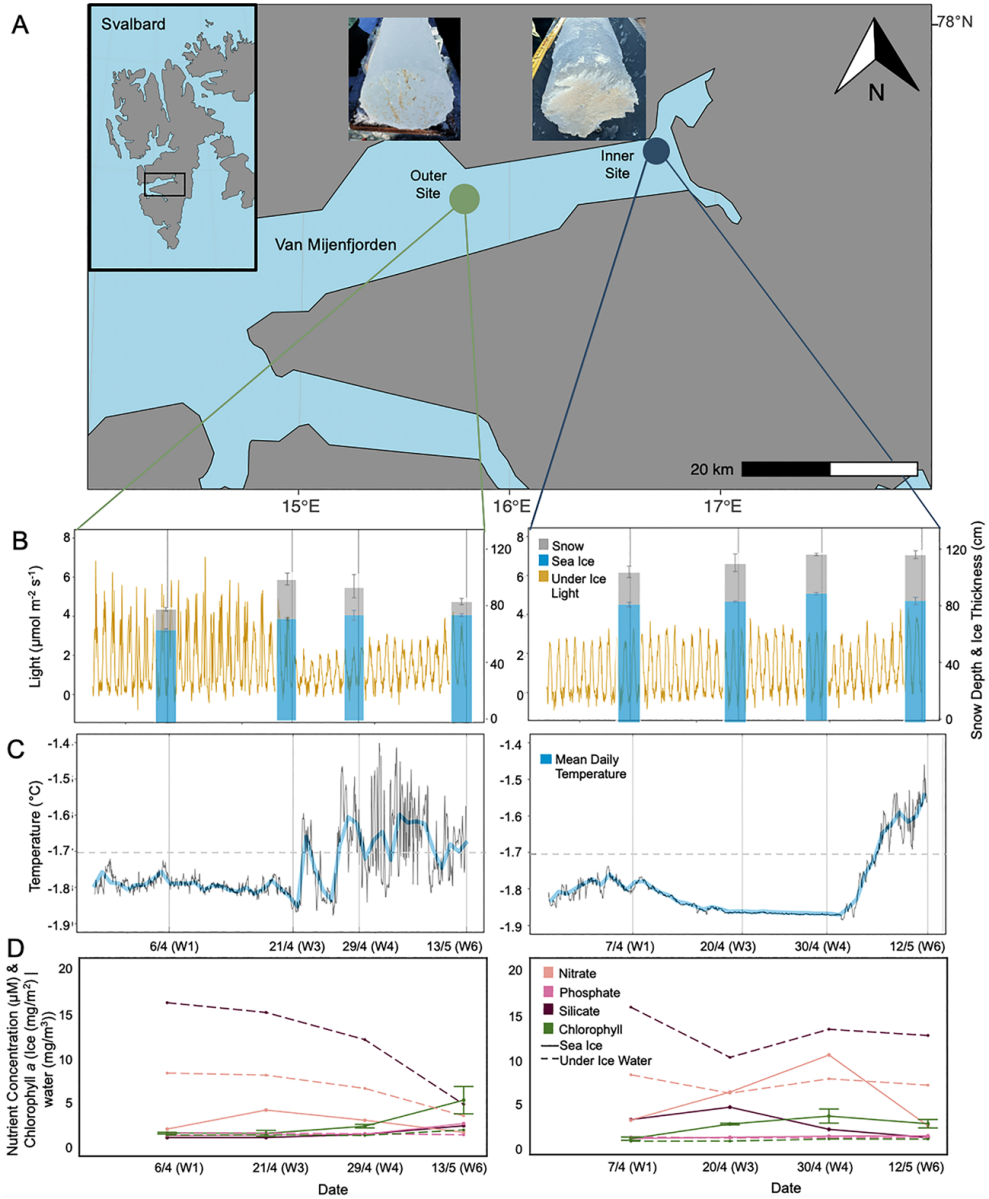
Location of sampling sites (**A**) visited between April–May 2022; inner site—blue dot and outer site—green dot, in Van Mijenfjorden, within Svalbard, Norway (inset box). Photographs of bottom ice cores collected from the inner and outer site on the 12.5.22 and 13.5.22 respectively, demonstrate more heterogeneity and lumping of biomass at the outer site. (**B**) Snow depth (cm) ± SD (grey bars), sea ice thickness (cm) ± SD (blue bars) and photosynthetically active radiation (PAR) (µmol photons m^−2^ s^−1^), measured by the light sensor deployed under the ice throughout the season (yellow line). (**C**) Under water temperature (black line) as measured by the temperature sensor deployed alongside the light sensor, solid line indicates average temperature (blue). (**D**) Nutrients and chlorophyll *a* concentration are displayed (bottom panel), including nitrate (light pink), phosphate (medium pink), silicate (dark pink) and chlorophyll *a* concentration (green) in the sea ice (solid line) and under-ice water (dashed line), on all sampling dates at the outer site (left) and inner site (right).

This study was conducted within Van Mijenfjorden in Svalbard, Norway (Fig. 1A) throughout April–May 2022. Two sites, one at the inner fjord (inner site; 77.80194 N, 15.76916 E) and one at the outer fjord (outer site; 77.84918 N, 16.7078 E), were sampled regularly (6–7.4.22; Week 1 (W1), 20–21.4.22; Week 3 (W3), 29–30.4.22; Week 4 (W4) and 12–13.5.22; Week 6 (W6)) to investigate seasonal changes in the biomolecular content of *N. frigida*, under different environmental conditions. Van Mijenfjorden is a partially enclosed fjord, with an inner basin approximately 70 m deep and an outer basin up to 120 m deep, located on the west coast of Spitsbergen, Svalbard, Norway^37^. The fjord experiences advection of warm, saline Atlantic water transported to the area by the West Spitsbergen Current (WSC), however this is substantially abated by the presence of the island Akseløya at the fjord mouth which allows for sea ice formation in winter. For further information on the conditions in Van Mijenfjorden see^19,30^. Sea-ice had been formed in the fjord since January, with closed drift-ice observed at the inner site from the 17.1.22 and from 24.1.22 at the outer site^38^.

### Sample collection

For each sampling event, six ice cores were extracted approximately 0.5 m apart, with a Kovacs core barrel (9 cm diameter; Kovacs Enterprise, Oregon, USA). The bottom 3 cm (at the ice-water interface) was retained, due to the microbial community being concentrated in this section of the ice^39,40^. The six ice cores were then pooled into triplicates, as cores 1–2, 3–4 and 5–6, and 100 mL of filtered sea water (FSW) (Glass Fibre Filter (GF/F), nominal pore size 0.7 µm) per centimetre of core was added to minimise osmotic stress^41,42^. The samples were then allowed to melt in darkness for 24 h at 4 °C. Cells were concentrated before biomolecular analysis; 100 mL from each of the three pooled samples was centrifuged at 1000 rpm (Universal 320, Hettich, Germany) for 4 min and the supernatant removed. The aliquot was then transferred to 2 mL Eppendorf tubes and centrifuged at 1000 rpm (Mikro 185, Hettich) for a further 2 min. The supernatant was again removed and the sample fixed by addition of formaldehyde (5% v/v) for later analysis. Additional cores were extracted for ice temperature (measured in situ), for bulk ice salinity (sectioned into 0–3 cm for bottom-ice and then 10 cm sections) and bulk ice nutrients (without the addition of FSW).

### Biological variables

#### Chlorophyll *a* content

Once the ice samples were completely melted, triplicate subsamples (150–400 mL) and a sample of under-ice water (500–1250 mL) were filtered (GF/F, 0.7 µm, Whatman, England) until visible colouration was observed on the filter. The filters were stored frozen (− 80 °C) until extraction and the extractions were performed within three months of collection. The analyses were performed according to^43^. Briefly, filters were extracted in 10 mL methanol and kept refrigerated at 4 °C for 24 h prior to extraction, after which Chl* a* content was measured with a calibrated Trilogy fluorometer (Turner, California, USA), before and after acidifying the extracts with 5% HCl^44^.

#### Community composition

From each sampling event, subsamples (100 mL) of the melted triplicate cores were collected into a brown bottle and preserved with glutaraldehyde (2% final conc.). Species composition was determined through the use of light microscopy; between 3 and 6 mL (depending on cell density) of the well-mixed subsamples were poured into an Utermöhl chamber with sedimentation cylinder (KC Denmark, Silkeborg, Denmark)^45^ and the cells were allowed to settle for up to 24 h^46^. Cells were counted at 400× magnification and identified to lowest possible taxonomic level. A whole chamber counting approach was employed to ensure rare taxa were captured. These analyses were conducted under a Nikon TS100 microscope within six months of sample collection.

#### Fatty acid composition

Samples from six pooled cores from each site on their final sampling date (inner site: 12.5.22 and outer site: 13.5.22) were filtered onto pre-combusted GF/F filters, until coloration was visible (300 mL from the outer site and 470 mL from the inner site). The filters were placed in glass vials with Teflon-lined caps and 8 ml of Methanol:dichloromethane was added and the vials were stored at − 80 °C until analysis. Total lipid was extracted according to Folch et al. 1957, at Akvaplan Niva AS, Norway.

### Environmental variables

#### Physical variables

Snow depth was measured three times in the close vicinity of the ice core extraction using a standard ruler, prior to core extraction. Ice thickness was measured at every core hole, after the core extraction, using a Kovacs ice thickness gauge. The ice temperature was measured in situ*.* Following complete melting of separate 10 cm sections, salinity of each section was measured (Thermo Scientific Orion Versa Star Pro). Based on these measurements, brine salinity and volume fractions were calculated according to Ref.^47^. A subsample of each pooled, melted ice cores was filtered onto pre-combusted GF/F filters until coloration was visible, for particulate organic carbon and particulate organic nitrogen (POC:PON) analysis. With each sampling event, a 0–3 cm section of an ice core was melted without the addition of FSW and then transferred to an acid washed bottle for bulk ice nutrient analysis. Also at each sampling event, approximately 100 mL of the water from directly below the ice-bottom surface was collected using a 5 L Niskin bottle (Hydro-Bios, Germany) and transferred to acid washed bottles for nutrient analysis. The nitrate plus nitrite (NO_3_^−^ + NO_2_^−^) (NO_x_), phosphate (PO_4_^3−^) and silicic acid (Si(OH)_4_) concentrations (µM) for ice and surface water were measured simultaneously on a QuAAtro 39 nutrient analyzer (SEAL Analytical, United Kingdom), with separate analysis channels for the three nutrients. The detection limits were 0.01 µM for NO_x_, 0.02 µM for phosphate and 0.02 µM for silicic acid. POC:PON analysis and stable isotope analysis on the bottom-ice and surface water was performed at the UC Davis Stable Isotope Facility in an EA-IRMS system, according to Ref.^48^. The detection limits were 100 µg C for δ^13^C and 20 µg N for δ^15^N.

#### Light measurements

At each sampling site, incoming photosynthetically active radiation (PAR) was measured using a LI-192 spherical underwater quantum sensor placed on a weighted frame moored ~ 1 m below the ice (from 26.3.22 until 13.5.22) recording PAR (400–700 nm) every minute (RBR*solo*^3^ PAR; RBR Global, Canada). Further, a temperature sensor (RBR*solo*^3^ T, accuracy of ± 0.002 °C) with every 0.5 min logger intervals was placed at the same depth. Albedo was calculated on each sampling date as snow reflected PAR, as proportion (%) of reflected PAR and incoming PAR to the snow surface, using a LI-190 quantum air sensor placed on the snow/sea ice surface to measure incoming PAR, and held level 1 m above the snow surface, facing the snow, to measure snow reflected PAR.

#### Species-specific biomolecular content by synchrotron-FTIR

The biomolecular composition of *N. frigida* was determined using synchrotron based FTIR microspectroscopy on formalin-fixed (5% v/v final concentration) cells. To ensure true replication, all cells measured were single cells and not dividing or associated with another cell. Samples were loaded directly into a micro-compression cell between two 13 mm diameter 0.5 mm thick BaF_2_ windows and spectral data of individual cells (x̄ = 26 cells per site, Table S1) obtained on the Infrared (IR) Microspectroscopy Beamline at the Australian Synchrotron, Melbourne. Each biomolecule absorbs a specific range of IR wavelengths, and a set of well-defined absorbance bands between 3050 and 2800 cm^−1^, and 1770–1100 cm^−1^ have been determined (Table 1). Spectra were acquired over the measurement range 4000–800 cm^−1^ with a Vertex 80v FTIR spectrometer (Bruker Optic, Ettlingen, Germany) in conjunction with an IR microscope (Hyperion 3000, Bruker) fitted with a narrow-band mercury cadmium telluride detector cooled with liquid nitrogen. To limit the light scattering effects, the measurements were performed on hydrated cells^49^. Co-added interferograms (sample n = 32, background n = 64) were collected at a wavenumber resolution of 4 cm^−1^. All measurements were made in transmission mode, using a measuring aperture diameter of 6.9 µm (area = 37.4 µm^2^). Cell compression was consistent ensuring the path length was uniform to account for any variation in cell size. To account for heterogeneity in the cell structure and distribution of biomolecules, all cells were measured with multiple points across the cell surface. Opus 8.0 software (Bruker) was used for both spectral acquisition and instrument control. Analyses were performed in July 2022 (within 6 months of samples being collected and fixed). All samples were kept refrigerated between fixation and analysis.

**Table 1:** Infrared (IR) band assignments for s-FTIR microspectroscopy used in this study.

Wave number (cm^−1^)	Band assignment	References
~ 3011	ν(CH)—from unsaturated fatty acids	^88^
~ 2852	ν_s_(C–H) from methylene (–CH_2_) from saturated lipids (CH-Stretch IV)	
~ 2921	ν_as_(C–H) from methylene (–CH_2_) from saturated fatty acids	
~ 1744	ν (C=O) ester carbonyl group from lipid triglycerides and fatty acids	
~ 1549	Protein (Amide II mode); mainly δ(N–H) of amides	^89^
~ 1400	ν_s_(COO^−^) from carboxylated molecules	^90^
~ 1241	ν_as_(PO_2_^−^) of the phosphodiester backbone of nucleic acids, phosphorylated proteins and lipids	^90,91^
~ 1191–1146	ν_s_(C–O) from carbohydrates	^92^
~ 1080	ν_s_(Si–O) from silica	^93,94^

#### Data analyses

Biomolecular content for each measured cell was determined by integrating the area under each assigned peak (Table 1), applying the Beer–Lambert Law assuming a direct relationship between absorbance and analyte concentration^50^. Data were smoothed (4 pts either side) and second derivative (3rd order polynomial) transformed using the Savitzky–Golay algorithm from the prospectr package^51^. Data were then normalised using Standard Normal Variate (SNV).

The community composition of all samples per site was contrasted by analysis of similarities (ANOSIM, Clarke & Warwick 1994), to determine if community composition was different between the inner and outer sites. Relationships between biomolecular content and sampling date and site were estimated using principal component analyses (PCA) and key biomolecular content per date was visualised using violin plots. To investigate which environmental variables account for a significant difference in the biomolecular content, redundancy analysis (RDA) was performed constrained to environmental variables (n = 6) with Monte Carlo permutations (999), following model optimisation, and only significant vectors displayed. Further redundancy analyses were performed, constrained to the stable isotopes (n = 4), to determine the effect on the differences in biomolecular composition. Regressions were tested for overall model significance of RDA analyses using the *F* statistic (*P* < 0.05) and strength of fit using R^2^. The residuals of all regressions were verified for homoscedasticity. The correlations between stable isotope of carbon (δ^13^C_VPDB_ (‰)) and lipid (ester carbonyl) and protein (amide II) content were visualised using boxplots. The Shapiro–Wilks^52^ test for normality showed that the biomolecular content required log_10_ transformation before analysis. All analyses were performed using RStudio v. 2023.09.463^53^ and the add-on packages ‘vegan’ v.2.6-4^54^, ‘ggplot2’ v.3.3.6^55^, ‘dplyr’ v.1.3.0^56^, ‘ggbreak’ v.0.1.2^57^.

## Results

### Physical and chemical environment

Under ice light at the outer site oscillated between 0 and 7 µmol/s/m^2^ until 21 April (W3), when it abruptly declined by ~ 50% (range: 0 and ~ 3 µmol/s/m^2^), a shift coincident with a doubling of snow depth from 15 ± 0.7 cm (W1) to 27 ± 2 cm (W2) (Fig. 1B). Albedo was highest (94%) on W3, increasing from 76% during early April (W1) (Table 2) due to changes in snow properties. For the inner site, under ice light remained constantly low (0–3 µmol/s/m^2^) for the entire sampling period, consistent with minimal changes in snow (x̄ = 27 ± 2 cm) and ice depth (Fig. 1B, Table 2). We measured abrupt increases in water temperature over the sampling period at both sites (Fig. 1C). However, for the outer site, under ice temperature reached expected sea ice melting point (> − 1.7 °C) in W3, followed by oscillations between freezing and thawing temperatures, whereas warming occurred after W4 at the inner site, with a steady increase to − 1.5 °C (Fig. 1C). Sea ice thickness gradually increased with time at the outer site, ranging from 64 ± 1 cm (W1) to 74 ± 1 cm (W6), and increased from 83 ± 2 cm (W1) to 90 ± 1 cm (W4) at the inner site, before declining to 85 ± 3 cm (W6). The bulk salinity in the bottom of the ice was similar between the two sites, ranging from 12.33 to 7.34 (x̄ = 10.1) at the outer site and 13.14–8.9 (x̄ = 11.2) at the inner site, with both sites experiencing the fresher conditions during W6 (Table 2). Brine volume did not vary between the two sites, ranging from 24 to 14% (x̄ = 20%) at the outer site and 28–19% (x̄ = 24%) at the inner site, with both sites experiencing the lowest brine volume during W6 (Table 2).

**Table 2:** Parameters measured associated with sea ice core extraction (white) and under-ice water (blue); snow depth (± SD, n = 18), albedo (%), ice thickness (± SD, n = 6), temperature (°C), bulk ice salinity (ppt), brine salinity (ppt), brine volume (% of ice volume), chlorophyll *a* concentration *(*Ice mg/m^2^, n = 3 and Water mg/m^3^), particulate organic carbon (POC) to particulate organic nitrogen (PON) ratio (C:N), stable isotope of carbon δ^13^C_VPDB_ (‰), stable isotope of nitrogen δ^13^N_VPDB_ (‰), silicic acid (Si(OH)_4_), nitrate + nitrite (NO_x_), phosphate (PO_4_) concentrations (µM) and average water temperature at the ice-water interface(°C).

Date	Station	Snow depth (cm)	Albedo (%)	Ice thickness (cm)	Ice temperature (°C)	Ice salinity (ppt)	Brine salinity (ppt)	Brine volume (%)	Chlorophyll *a* (mg/m^2^|mg/m^3^)	C:N	δ^13^C_VPDB_ (‰)	δ^15^N_VPDB_ (‰)	Si(OH)_4_ (µM)	NO_3_^−^ + NO_2_ (µM)	PO_4_ (µM)	Average water temperature (°C)
6.4.22 Week 1	Outer	15 ± 0.7	76	64 ± 1	− 2.3	10.80	40.78	24	0.52 ± 1	6.14	− 25.01	3.81	N.D	0.98	0.24	
									*0.29*	*4.95*	− *31.31*	*2.04*	*15.61*	*7.46*	*0.51*	− *1.79*
7.4.22 Week 1	Inner	22 ± 2	95	83 ± 2	− 2.1	9.74	37.36	23	0.31 ± 1.9	2.75	− 26.81	3.26	2.51	2.46	0.34	
									*0.02*	*3.99*	− *28.91*	*2.56*	*15.37*	*7.62*	*0.51*	− *1.79*
20.4.22 Week 3	Inner	27 ± 4	77	85 ± 0.5	− 2.6	13.14	45.85	26	2.00 ± 0.1	5.40	− 21.95	2.49	3.93	5.65	0.47	
									*0.04*	*3.16*	− *26.5*	*2.18*	*9.62*	*5.52*	*0.40*	− *1.87*
21.4.22 Week 3	Outer	27 ± 2	94	70 ± 1	− 2.7	12.33	47.53	23	0.49 ± 0.3	2.62	− 27.14	1.48	N.D	3.19	0.30	
									*0.31*	*2.57*	− *26.86*	*4.69*	*14.48*	*7.22*	*0.53*	− *1.85*
29.4.22 Week 4	Outer	19 ± 5	90	73 ± 3	− 2.5	9.78	44.15	19	1.32 ± 0.2	4.79	− 22.65	2.88	0.38	2.01	0.40	
									*0.25*	*5.67*	− *24.83*	*4.03*	*11.33*	*5.70*	*0.45*	− *1.65*
30.4.22 Week 4	Inner	28 ± 0.5	93	90 ± 1	− 2.3	13.00	40.78	28	2.91 ± 0.8	3.24	− 22.92	0.96	1.38	9.90	0.60	
									*0.28*	*4.85*	− *26.08*	*3.49*	*12.84*	*7.17*	*0.41*	− *1.87*
12.5.22 Week 6	Inner	32 ± 1.5	78	85 ± 3	− 2.3	8.90	40.78	19	2.03 ± 0.5	4.04	− 23.41	3.05	0.45	1.79	0.65	
									*0.27*	*7.15*	− *24.73*	*4.25*	*12.13*	*6.44*	*0.47*	− *1.50*
13.5.22 Week 6	Outer	9 ± 1	88	74 ± 1	− 2.6	7.34	45.85	14	4.33 ± 1.6	11.51	− 16.29	4.24	1.37	0.60	1.62	
									*0.88*	*6.33*	− *25.17*	*4.6*	*3.86*	*2.52*	*0.31*	− *1.67*

Ice NO_x_ concentrations peaked (3.2 μM) in W3 at the outer site, before declining to potentially limiting levels (0.6 μM) by W6. In contrast, NOx concentrations were higher at the inner site (averaging 5.0 ± 3.7 μM throughout the study), with a peak concentration of 9.9 μM in W4 (Fig. 1D). For the outer site, Si(OH)_4_ concentration in the ice was not detected until W5 when it was low (0.4 μM), while PO_4_^3−^ increased to 1.6 μM in W6 (Table 2). Unlike the outer site, ice Si(OH)_4_ concentrations were consistently detectable at the inner site, with the concentration ranging from 0.5 to 3.9 μM (Fig. 1D). Chl* a* concentrations within the bottom ice gradually increased with time at both sites, peaking at W6 for the outer site and W4 for the inner site (Fig. 1D; Table 2). There was a visible change in the chl* a* biomass within the bottom-ice following the melting period at the outer site (Fig. 1D), in which the biomass was heterogenous and forming visible lumps. Pelagic chl* a* concentrations remained low throughout, increasing to 0.9 and 0.3 mg/mg^3^ by W6 at both the outer and inner site, respectively (Table 3; Fig. 1). Seawater NO_x_ and Si(OH)_4_ concentrations decreased with time at the outer site, but remained replete (NO_x_: 2.5–7.5 and Si(OH)_4_: 0.9–3.8 µM, respectively). PO_4_^3−^ concentration remained constant (0.3–0.5 μM) and replete. For the inner site, concentrations of NO_x_ and Si(OH)_4_ and PO_4_^3−^ in the seawater remained relatively constant and replete (NO_x_: 6.4–7.6 μM, Si(OH)_4_: 9.6–15.4 μM and PO_4_^3−^: 0.4–0.5 μM) throughout the study (Fig. 1D). Molar ratios of C:N did not differ between sites (Table 2). However, there was a greater range in C:N (2.62–11.51) at the outer site than the inner site (2.75–5.40). Molar ratios of N:Si taken from the ice ranged between 0.44 and 5.29 at the outer site and 0.98 and 7.17 at the inner site, whilst the molar ratio of Si:P ranged between 0.85 and 0.95 at the outer site and from 0.70 to 8.36 at the inner site (Table 2). Bottom-ice POC and Chl *a* were found to be positively correlated at the outer site only (*F*_1,2_ = 51.72, p < 0.05, R^2^ = 0.96) and POC:Chl *a* did not exhibit a relationship with time at either site.

**Table 3:** Fatty acid (FA) composition of the entire sea ice algae community at the inner and outer site of Van Mijenfjorden, Svalbard on the 12th May and 13th May 2022, respectively, as % total FA displayed (with SUM % total lipid dry matter (DM) displayed in brackets), for 6 ice cores pooled. FAs accounting for < 0.1% of total in both sites are not displayed. FA: Fatty acids, PUFA: polyunsaturated fatty acids, MUFA: monounsaturated fatty acids, SAFA: saturated fatty acids, EPA: eicosapentaenoic acid and DHA: docosahexaenoic acid.

	Outer site	Inner site
	% FA	% FA
14:0	7.5	5.5
15:0	0.3	0.4
16:0	20	19
16:1 n-9	2.7	8.5
16:1 n-7	49	22
16:1 n-5	0.6	1.5
17:0 Phytanic	0.1	0.4
17:0	0.1	0.5
16:2 n-7	0.9	1.9
16:3 n-4	0.5	1.9
18:0	1.4	6.2
16:4 n-1	1.4	3.1
18:1 n-9	1	1.5
18:1 n-7	0.3	0.7
18:2 n-6	0.6	0.6
18:3 n-6	1.9	1
18:3 n-3	0.3	0.4
20:0	0.2	0.4
18:4 n-3	1.4	2.1
20:4 n-6	0.8	3.4
20:3 n-3	0.2	0.3
22:0	0.1	0.4
20:4 n-3	0.2	0.3
22:1 n-9	0.4	3.3
20:5 n-3	6.9	10
22:6 n-3	0.9	2.1
SUM FA of DM	52	25
SUM SAFA	30 (16)	35 (8.6)
SUM MUFA	54 (28)	38 (9.4)
SUM PUFA	16 (8.4)	28 (6.8)
SUM omega-3 FA	10 (5.2)	16 (3.9)
SUM omega-6 FA	3.3 (1.7)	5 (1.3)
SUM omega-9 FA	4.1 (2.1)	13 (3.3)
SUM EPA + DHA	7.8 (4.1)	13 (3.1)

### Protist community composition

**Figure 2:**
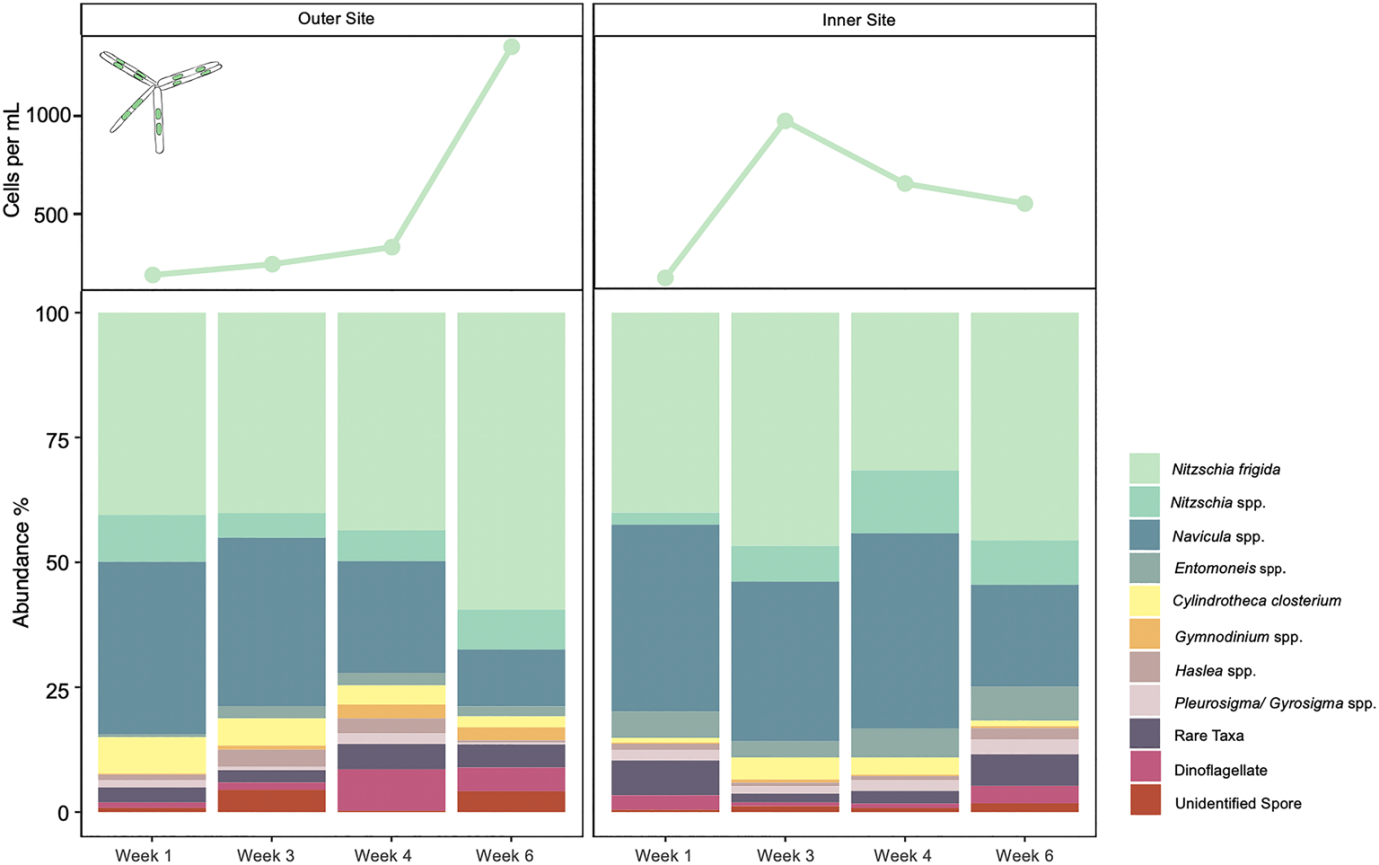
Bottom ice microalgal community composition from outer site (left) and inner site (right) sampled during April–May 2022, in Van Mijenfjorden, Svalbard, Norway. Raw counts of *Nitzschia frigida* cells per mL are displayed in the top panels, and the relative abundance (%) of taxonomic groups are shown in the lower panels. *Nitzschia* spp. includes all *Nitzschia* species identified with the exception of *Nitzschia frigida*. Rare Taxa includes: *Hantzchia* spp., *Diploneis littoralis*, *Stenoneis inconspicua*, *Pinnularia quadratarea*, *Plagiotropis* spp. *Manguinea rigida*, *Tropidoneis* spp.

*Nitzschia frigida* was the most abundant taxa at all sites and all time points, making up between 31% of the community at the inner site during W4 and 56% at the outer site during W6 (Fig. 2), with an increase in abundance towards the end of the season. Sea ice community composition was similar across the two sites and all time points (ANOSIM R = 0.03, p > 0.05), although some apparent decline in the proportion of *Navicula* spp. and *Pleurosigma/Gyrosigma* spp, in conjunction with an increase in the proportion of dinoflagellates, including *Gymnodinium* spp. is evident at the outer site by W6 (Fig. 2).

### Biomolecular composition

**Figure 3:**
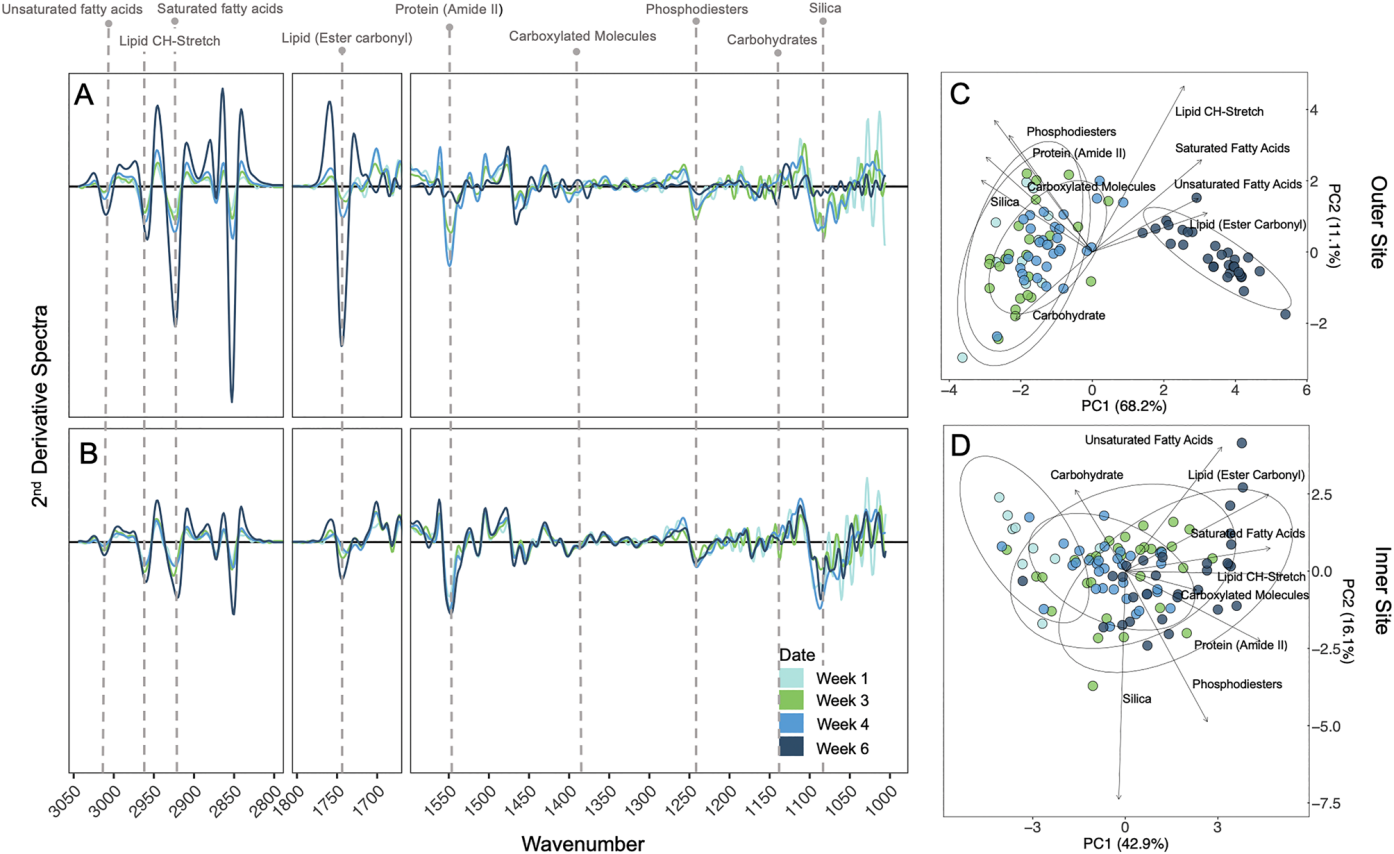
Smoothed and normalised 2nd derivative spectra for *Nitzschia frigida* at the (**A**) outer and (**B**) inner site with each sampling week denoted by colour gradient. Dashed vertical lines indicate respective wavenumber for peaks of interest. Principal component analysis (PCA) of biomolecular content at the (**C**) outer and (**D**) inner site with sampling week denoted by colour, in which each dot represents the measurements of one cell. Direction and strength of individual biomolecules are displayed with ordination bi-plot overlay.

Spectral analyses revealed strong trends and differences in key biomolecular peaks across time for both sites (Fig. 3A,B). Principle component analysis on the nine selected biomolecular peaks of *N. frigida* from the outer site showed separation with time of sampling along PC1, explaining 68.2% of the variation (Fig. 3C). The temporal trend was primarily driven by relative increases in lipids and FAs at the last sampling time point (W6). For the inner site, PC1 explained 42.9% of the variation, with evidence of a temporal effect driven by increases in lipid and saturated FAs (Fig. 3D).

**Figure 4:**
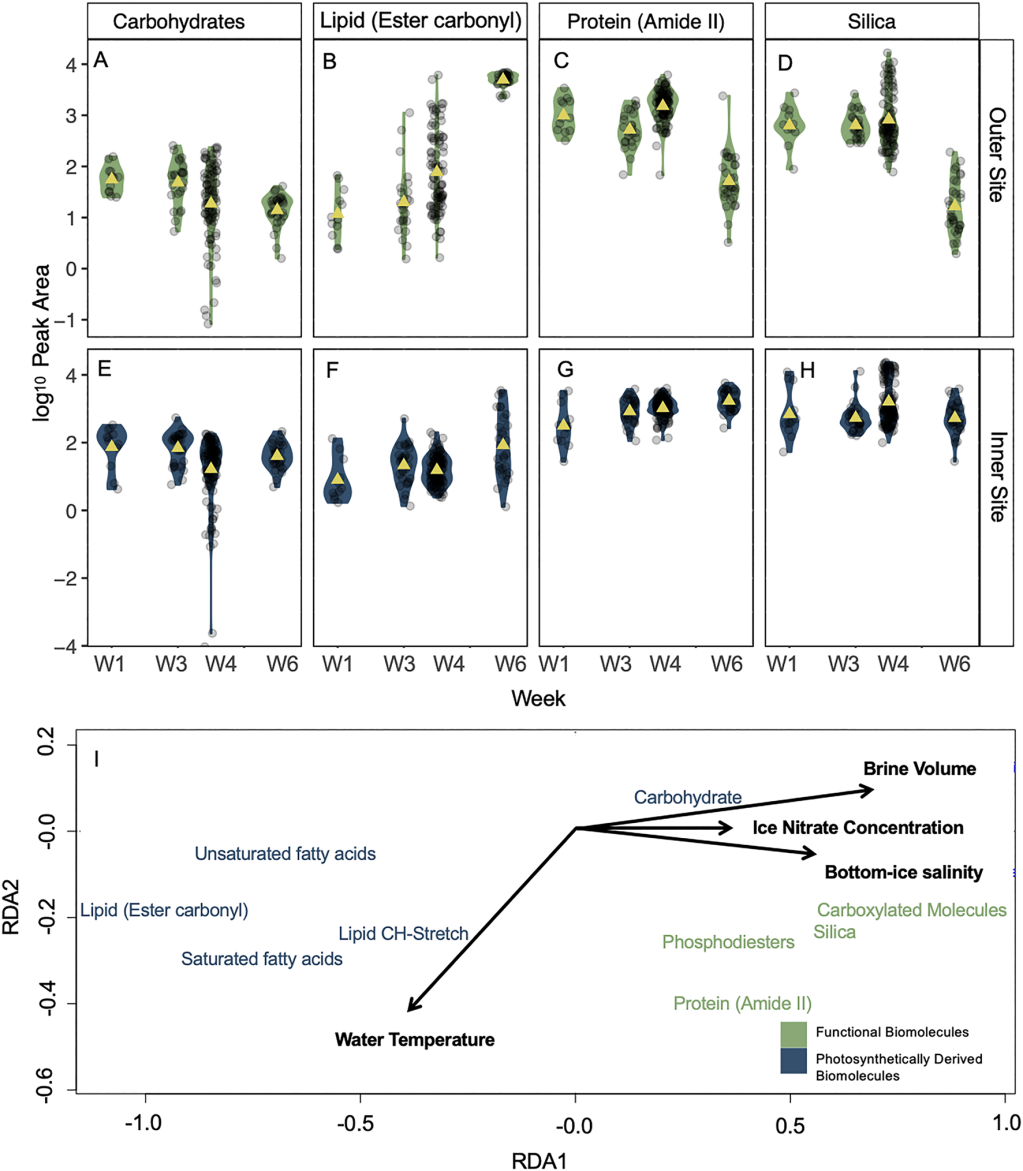
Biomolecular content based on normalised peak areas of specific biomolecules, for *Nitzschia frigida* per sampling week for the outer (**A**–**D**) and inner site (**E**–**H**); specifically (**A**, **E**) Carbohydrate (**B**, **F**) Lipid (Ester carbonyl), (**C**, **G)** Protein (Amide II) and (**D**, **H**) Silica content. Data are presented as violin plots where coloured shading indicates SE and a yellow triangle represents the mean, individual cells are shown by transparent dots. Site location is represented with colour; outer site (green; top panels) and inner site (blue; bottom panels). Redundancy analysis (RDA) biplot (**I**) of the mean biomolecular content (divided into functional biomolecules (cell structure and function) and photosynthetically derived biomolecules (energy and storage), denoted by colour) from each sampling site and date with environmental variables displayed with ordination bi-plot overlay. Only significant vectors are shown and RDA model is significant (p < 0.05).

Integrated peak areas for selected biomolecules revealed high cell-specific variability in *N. frigida* from both sites (Fig. 4A–D). We detected a significant shift in biomolecular composition of *N. frigida* at the end time point for the outer site (Fig. 4A–D), where differences observed were driven by an increase in allocation to storage biomolecules i.e. lipid (ester carbonyl) (*F*_1,93_ = 148.6, p < 0.05, R^2^ = 0.62; Fig. 4B), saturated fatty acids (SFA) and unsaturated fatty acids (USFA) (Table S2). These increases were concomitant with a decrease in protein (Amide II) (*F*_1,94_ = 60.16, p < 0.05, R^2^ = 0.39; Fig. 4C), carbohydrates (*F*_1,93_ = 26.54, p < 0.05, R^2^ = 0.22; Fig. 4A), phosphodiesters and carboxylated molecules (Table S2). In contrast, *N. frigida* cells from the inner site, showed a significant shift in biomolecular profiles from early April (W1) and the final time point (Fig. 4E–H), primarily driven by a relative increase in protein (Amide II) (*F*_1,100_ = 44.19, p < 0.05, R^2^ = 0.31; Fig. 4G) and decrease in carbohydrate content (*F*_1,100_ = 44.19, p < 0.05, R^2^ = 0.31; Fig. 4E). A significant increase in lipids (ester carbonyl) (*F*_1,99_ = 26.99, p < 0.05, R^2^ = 0.21; Fig. 4F) and SFAs were also observed (Table S2). In addition to interrogating the biomolecules in the cell, we also looked for any changes in the silica peak at 1150 cm^−1^. Unexpectedly, we saw a significant decline in biogenic silica in cells from the outer site by W6 (Fig. 4D), corresponding with the other significant changes in biomolecular components. No change in silica content was detected for *N. frigida* cells from the inner site (Fig. 4H).

Redundancy analysis revealed that the environmental parameters of brine volume, bottom-ice salinity and ice nitrate concentration combined explained 77% of the variability in biomolecular content across the two sites, while seawater temperature explained 11% of the variability (*F*_6_ = 2.92, p < 0.05, Fig. 4E). Photosynthetically derived biomolecules (e.g. lipids and FAs) were negatively correlated with brine volume, bottom-ice salinity and ice nitrate, yet showed a positive relationship with these environmental parameters for the functional biomolecules (e.g. protein, phosphodiesters and silica content) and carbohydrate content. Water temperature (i.e. freezing or melting the bottom of the sea ice) was shown to have the greatest positive effect on lipid (CH-stretch) content and an inverse relationship with carbohydrate content (Fig. 4E). Previous studies have found increasing light to drive increases in lipid and FA allocation in sea ice algae^19,58,59^ but the typical increase light transmitted to the bottom-ice toward the end of the productive season was not observed in this study, excluding light as a significant driver in determining biomolecular changes.

### Fatty acid composition

**Figure 5:**
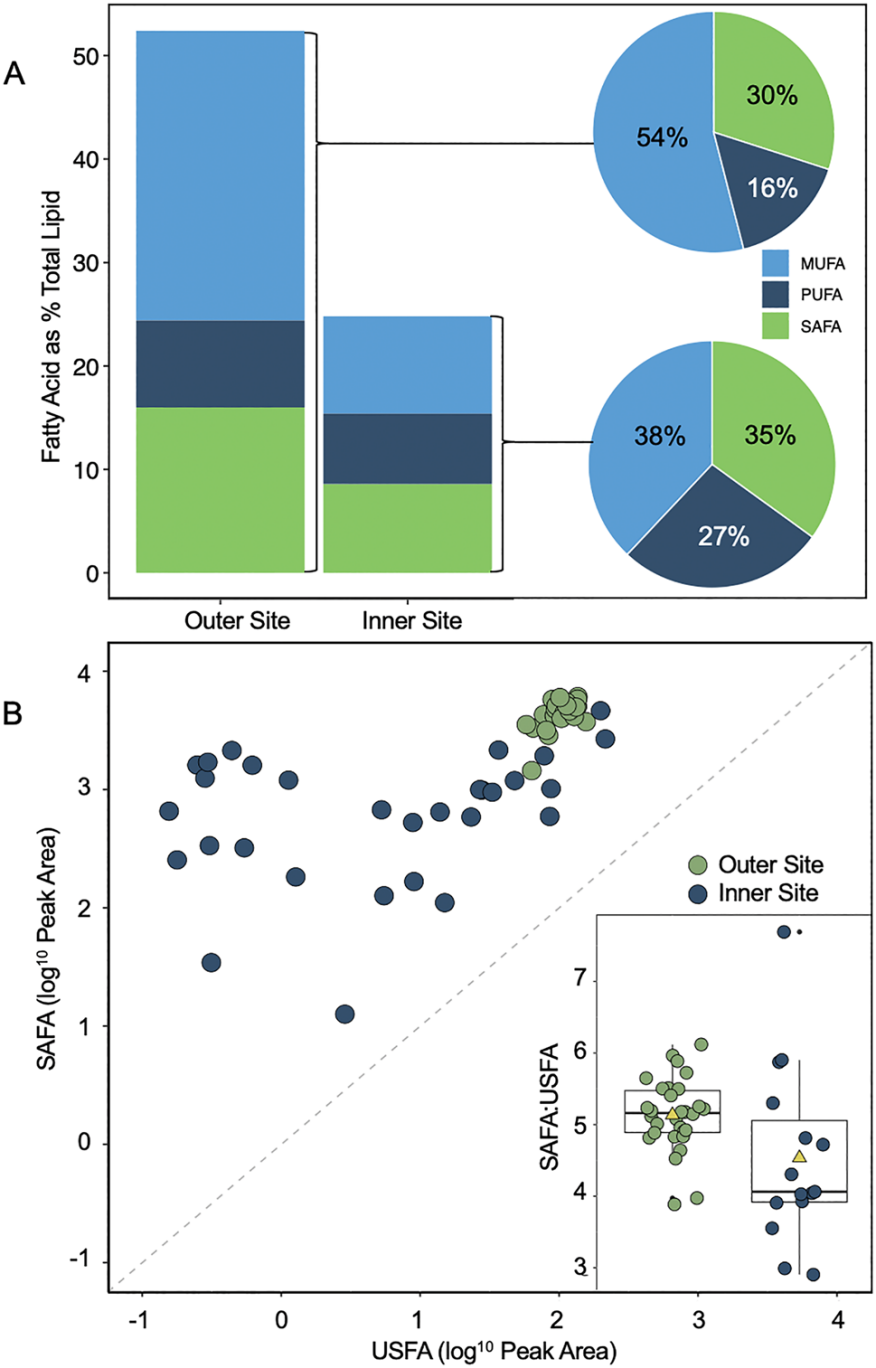
Fatty acid (FA) content as % total lipid content of the whole bottom ice community at the outer and inner site (**A**), during week 6. The different FA types, monounsaturated fatty acid (MUFA), polyunsaturated fatty acid (PUFA) and saturated fatty acid (SAFA) per site, are denoted by colour. Site-specific FA content as a proportion of total FA are presented in pie charts (right). Cell-specific SAFA vs. unsaturated fatty acid (USFA) content of *Nitzschia frigida*, as determined by s-FTIR (**B**), with site denoted by colour. The ratio of SAFA:USFA content per cell is displayed (inset), as a boxplot with yellow triangles representing the mean.

Total fatty acid (FA) content of the outer site community was double (54% total lipid of dry matter) that of the inner site community (25% total lipid of dry matter) (Fig. 5A). The predominant FA at both sites was 16:1 n-7, followed by 16:0, 20:5 n-3 and 14:0 (Table 2), together accounting for 83.5% and 56.5% of FA content at the outer and inner sites, respectively. As a proportion of total FA content, both sites had a similar relative proportion of SAFA content (30% FA: inner site, 35% FA: outer site). However, the inner site had a relatively higher proportion of PUFA content (28% FA: inner site, 16% FA: outer site) and a lower proportion of MUFA content (38% FA: inner site, 54% FA: outer site) compared with the outer site (Fig. 5A). In addition, the proportion of omega-3 FA EPA + DHA (polyunsaturated eicosapentaenoic acid and docosahexaenoic acid) was almost double at the inner site (13% FA) compared to the outer site (7.8% FA). Comparing SAFA and USFA content from the total community analysis with the FTIR samples from the final time point, we see that in both *N. frigida* and the total community, the total FA content was substantially higher at the outer site (Fig. 5B). There was a strong relationship between SAFA and USFA content at the outer site (*F*_1,28_ = 16.35, p < 0.05, R^2^ = 0.37) but not at the inner site (*F*_1,28_ = 2.4, p > 0.05, R^2^ = 0.08), where more variability was observed (Fig. 5B).

### ***δ***^***13***^*** and δ***^***15***^***N stable isotopes***

**Figure 6:**
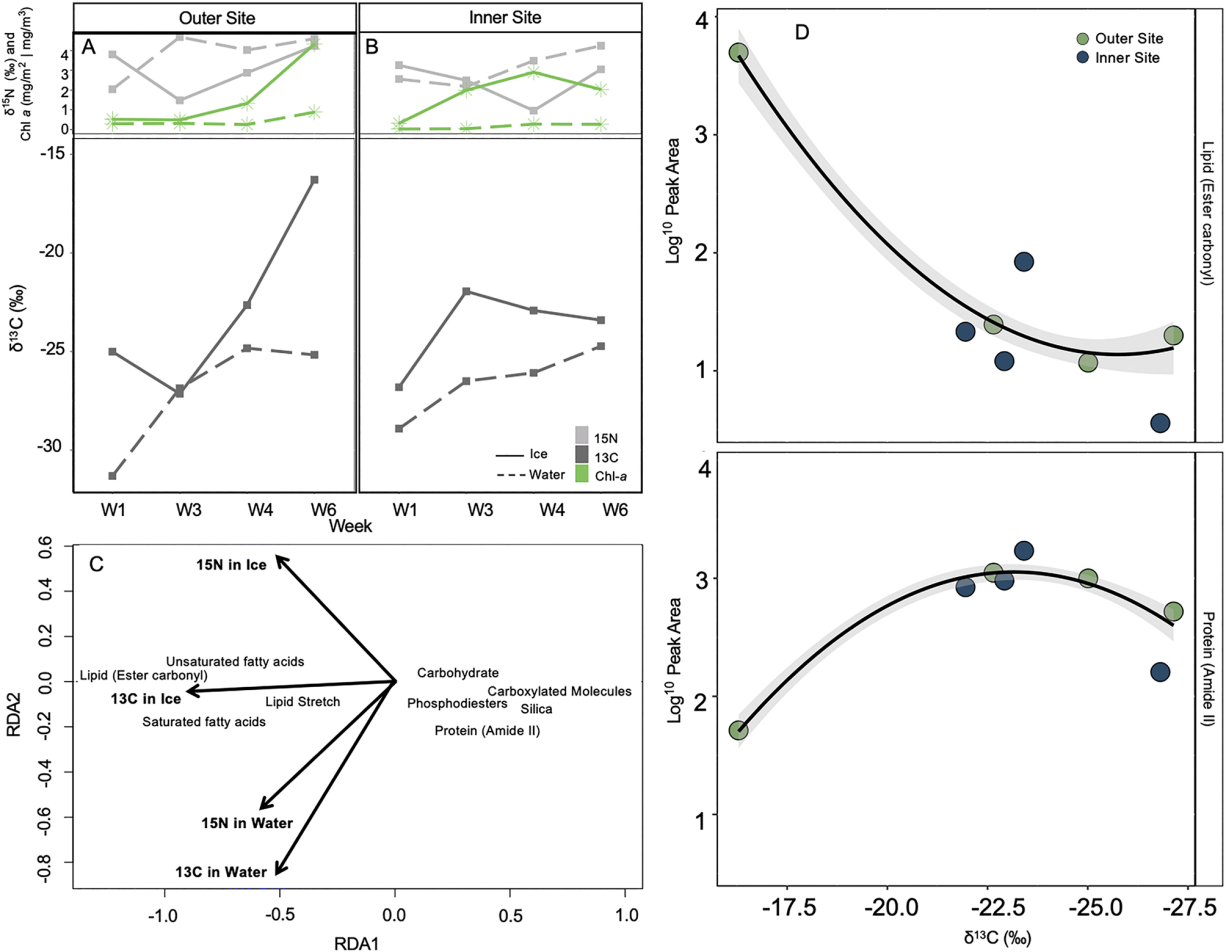
δ^15^N (light grey square), δ^13^C (dark grey square) and chlorophyll* a* content (green star) from the bottom-ice (solid line) and water directly below ice (stipple line) at the (**A**) outer site and (**B**) inner site, across all sampling weeks. Redundancy analysis (RDA) biplot (**C**) of the mean biomolecular content of *N. frigida* cells from each sampling site and time point with δ^13^C and δ^15^N stable isotope values from the sea ice and water below displayed with ordination bi-plot overlay, only significant vectors are shown and RDA model is significant (p < 0.05). (**D**) Content of lipid (ester carbonyl; top panel) and protein (amide II) (bottom panel) vs. δ^13^C values, at the outer site (green) and inner site (blue). The data (both locations combined) are fitted with linear regressions, with 95% confidence intervals (grey shading).

Neither ice nor seawater δ^15^N_Air_ (‰) differed between sites, and showed no distinct pattern nor relationship to chl* a* concentrations (Fig. 6A,B). Conversely, under-ice seawater at both sites, and ice at the outer site, showed increased carbon-enrichment over time, with δ^13^C_VPDB_ (‰) ranging from − 31.31 to − 25.17 ‰ in the water and − 25.01 to − 16.29 ‰ in the ice at the outer site (Fig. 6A,B). Unlike the δ^15^N values, the δ^13^C values from the ice mirrored the patterns observed in the ice chl* a* concentration (Fig. 6A,B). There was no significant difference in carbon enrichment between the sites for the ice or under-ice water (Fig. 6A,B). Carbon enrichment was positively associated with storage biomolecular content (i.e. lipid and FAs), and inversely related to protein, carbohydrate, silica, carboxylated molecule and phosphodiester content (Fig. 6C). These relationships were driven by changes at the outer site only, where peak carbon enrichment was associated with the highest lipid content and lowest protein content (Fig. 6D).

## Discussion

Our study followed the seasonal progression in biomolecular composition of the key sea ice diatom, *N. frigida*. By sampling from two locations at four separate time points spanning five weeks during spring, we were able to capture spatial and temporal dynamics in environmental conditions from an inner (more stable) and an outer (more dynamic) fjord site and look at them in relation to plasticity in biomolecular properties. Furthermore, by analysing individual cells we revealed high plasticity and individual variability in *N. frigida* cell content, with major changes becoming evident with the onset of seasonal melt. Close examination of the biomolecular profiles in relation to environmental variables, revealed that nitrogen limitation, in conjunction with warming sea temperatures and decreased brine volume, corresponded with increased lipid and FAs stores, and a decline in protein and carbohydrate. These shifts in metabolic energy allocation may be representative of the preparation for over wintering in *N. frigida* cells, as the Arctic summer advances.

Increasing ocean temperature is a key environmental trigger for initiating the seasonal transition from spring into Arctic summer. There was a two-week difference in the timing of warming between our two sites, making evident the influence ice melt can have on *N. frigida* populations. Throughout the study, the community was dominated by diatoms (predominantly *N. frigida*). Our microscopy findings were supported by the FA composition analyses, which showed 20:5 n3, C16 PUFAs and 16:1 n7—the diatom-marker FAs^60^ as the primary constituents. From the onset of under-ice melt, we measured an increase in *N. frigida* numbers*,* as well as an increase in the proportion of dinoflagellates at the outer site, mirroring trends previously observed with seasonal progression^30,61–63^. Further, at the outer site with slightly younger ice, the under-ice melt resulted in a decrease in the proportion of *Navicula* spp. and increase in unidentified spores during mid-April (W3). The early warming at the outer site (starting W3), was followed by a series of temperature oscillations between freezing and thawing until mid-May (W6), causing physical changes to the bottom of the sea ice (i.e. removal of skeletal layer) and a reduction in brine volume (i.e. space for community colonisation). Interestingly, despite the loss of skeletal layer and reduction in brine volume from W4 onwards, the chl-*a* content within the ice increased at the outer site, forming a patchy distribution of biomass, still dominated by *N. frigida.* This ability for *N. frigida* to grow as large aggregates, likely provides a competitive advantage to *N. frigida* as it forms dendroid colonies, allowing it to remain on the bottom-ice, even in the absence of a skeletal-ice layer and expansive brine channel network. In addition, *N. frigida,* along with many other pennate diatoms, can produce exopolysaccharides (EPSs), creating a viscous biofilm and increasing cell adhesion to the bottom-ice^64^. These adaptations explain the sympagic biomass increase despite less advantageous sea ice conditions. The predominance of such aggregates has implications for secondary production and carbon export^65^ with zooplankton unable to graze on larger food packages as effectively^66^ and the aggregates falling to the sea floor more quickly^67^, enhancing carbon deposition.

Our single-celled approach revealed high intra-specific variability in biomolecular content, suggesting that *N. frigida* possess many plastic traits able to adjust to the dynamic spring sea ice and water conditions, providing a plausible evolutionary explanation for its ecological success in the sea ice and dominance throughout the entire productive season (Figs. 3, 4). We also detected considerable reduced variability in expressed lipid content at the last time point for the outer site, which could be indicative of strong environmental forcing causing cellular stress, whereby cells reach their phenotypic limit, in this case maximum production and storage of lipids. These changes coincided with significant decrease in protein and silica, supporting the idea of possible stress-induced changes in metabolic homeostasis. This type of latent stress response is often typical of variations in temperature^21^, such as the observed warming, reduced nitrogen availability^23^, and/or salinity^68^.

Alongside warming, nitrogen depletion, following its consumption by primary production, often signals the end of the productive ice-algae season^62,69^, and typically occurs with the beginning of the ice-breakup. As such, microalgal cells respond to nitrogen limitation by increasing lipid stores in preparation for dormancy^22,59^. Therefore, the rapid and significant increase in lipid accumulation by *N. frigida*, could be indicative of reduction in nitrogen availability^70^. The Redfield ratio for sea ice algae (amended to include the silicate requirement for diatoms) is generally accepted to be 106C:16N:15Si:1P^44^. Based on these requirements, in our study, nitrogen at the outer site could only have become limiting in mid-May, coinciding with the strongest biomolecular changes. The relatively constant and replete nitrogen levels within the other samples was likely due to exchange with seawater below the ice^13^, and/or remineralisation and nitrification^71,72^ within the ice. Nitrate replenishment is indicated by low δ^15^N values and the relatively low chl* a* content and biomass, supported by the generally low C:N values (indicating low carbon), observed at the other sampling points throughout the season. Another indication of possible temperature and nitrogen stress was the observed decline in cellular energy allocation to protein and reduced carbohydrate content, as such shifts have been previously observed in nitrogen limited sea ice algal communities^22,23,59^. The biomolecular shifts in energy allocation toward lipid storage, suggest that under warmer, nitrogen limiting conditions, further growth is not prioritised.

While the inner site showed little variation over our seasonal sampling, the outer site community was found to have approximately double the total FA content than the inner site, with proportionally higher MUFA content on the final sampling date. This is consistent with increases in neutral lipids under nitrate limitation, and as primarily storage lipids, this demonstrates a response to stress and preparation for dormancy^73,74^. In addition, the outer site had substantially higher 16:1 n7 content, an indicator of the cell entering a storage and survival phase. The community from the inner site on the final sampling date was indicative of a community in exponential growth with proportionally more PUFAs (and specifically higher proportions of 20:5 n-3 and 16:4 n-1)^75^, and which is consistent with nitrate limited cells taken post-bloom^60,75,76^. The availability of PUFA content has been shown to determine zooplankton production and growth^16,77,78^. The omega-3 FAs (PUFAs) EPA and DHA are essential for growth and reproduction in all marine organisms and are key indicators of how nutrient rich a food source is, yet are produced exclusively by marine algae^79^. Furthermore, the increase in lipid observed in *N. frigida*, corresponding with an increase in total carbon enrichment (δ^13^C) within the ice^40^, indicates that the biomolecular response of *N. frigida* could be representative of many taxa in the sea ice algae community. Taken together, the species-specific spectroscopy and community FA composition and carbon enrichment results from the outer site elucidate how end of season nitrate limitation alters the nutritional quality of food available to higher trophic levels.

In response to seasonal warming, another environmental change signalling the end of the ice season is a decrease in brine volume and associated freshening of the bottom-ice. While this process can occur during ice growth, drainage is often triggered by either warming at the ice surface (air temperature), or melting at the ice-water interface (water temperature)^80^. The hypersaline brine drains out to the ocean below and fresh meltwater from the ice percolates through the brine network, replacing the brine^81^ and lowering interstitial salinity. In this study, we measured a decrease in brine volume and freshening of ice at the outer site on the last sampling time point, coinciding with detected changes in biomolecular profiles. Whilst the bulk ice salinity was within expected ranges for the end of the ice season (e.g.^62,68,82^) and brine volume remained above the 5% threshold necessary for colonisation^83^, the changes appeared to be sufficient to create environmental stress for the *N. frigida* cells. The move toward hypoosmotic conditions, which have been found to significantly lower photosynthetic efficiency in pennate ice diatoms^68^, could have induced strong increases in lipid storage and decreases in carbohydrate production as a means to prioritise energy storage over growth and development, in readiness for a period of dormancy^22,74^. Such adaptations to seasonality are commonly observed in many animals^84^, but are to a lesser degree documented for phytoplankton. Zooming in on individual properties of microalgae cells, as done in this study, adds much power when aiming to identify the extent of such adaptations in diatoms and other single cell organisms in the ocean. At the same time, the decrease in carbohydrate content and increase in lipid could be attributed also to the enduring higher average water temperatures (− 1.65 °C, a temperature expected to melt sea ice from below) from the end of April. Similar biomolecular changes have been observed previously, albeit over a greater temperature change, e.g. between − 1.8 and + 3 °C^21^. This is the first time such changes have been reported at the boundary temperature between melting and freezing. Given the above, it is likely that the combination of a decrease in brine volume, freshening of the bottom-ice and warmer under-ice water temperature contributed to creating a multi-stressor environment, and when combined with nitrogen limitation, directed metabolism and photosynthate allocation toward increased energy storage rather than continued growth and development.

Unlike nitrate, silicate was limiting at both sites throughout the season, with N:Si higher than Redfield (1.07) and Si:P lower than Redfield (15) at both sites^85^. As silicate is necessary for building and maintenance of diatom frustules^86^, under limitation, biogenic silica formation and therefore diatom growth, is constrained^87^. Our data showed a strong decline of biogenic silica in *N. frigida* at the final time point, which may be explained by the additional stress on the cells experiencing end of season conditions, compromising their growth and frustule formation.

The results from this study demonstrate how environmental conditions that lead to the sea ice algae community being ‘released’ from the ice, are important in terms of the cell’s viability for surviving ice free conditions, for carbon export, and for energy supply to the marine food web. With the onset of warming, the biggest environmental changes to influence *N. frigida* physiology, morphology and metabolism, were reduced brine volume and the onset of nitrogen limitation. The sea ice algal community, and *N. frigida* in particular, showed high phenotypic diversity and plasticity throughout the sea ice season, and appeared to ‘tolerate’ the dynamic freeze/thaw cycles, continuing to grow, independently or perhaps because of the variable conditions. Variability in the phenome of *N. frigida* was reduced as the season progressed towards ice-free conditions. We saw large changes in biomolecular composition, specifically an increase in lipid and FA storage, at the expense of protein and carbohydrate stores, associated with the onset of nitrogen limitation, marking the end of the sea ice algal growth season. As lipid stores and reduced metabolic rate are thought to be the key to the ability of *N. frigida* to survive summer and the six months of darkness that follow, these findings highlight the importance of this seasonal metabolic cascade for Arctic food web dynamics and carbon export. Our study revealed a significant decline in cell-specific silica content as the community approached the end of the cascade. This reduction in cell-specific silica content, has the potential to alter *N. frigida* sinking rates and grazability, thereby influencing carbon transfer. Conversely, these changes may be countered by the increased aggregation by *N. fridiga*, which would likely result in a net increase in population sinking rate and provide additional protection from potential grazers. In the context of climate change, the predicted Atlantification of the Arctic would bring warmer water advection earlier in the season and/or create a more abrupt transition to the melting phase. This transition earlier in the season could obviate these important end-of-season environmental shifts, causing the algae to be released from the ice prior to significant biomass accumulation and sufficient lipid build-up and storage, with the onset of nitrogen limitation. Reduced biomass would likely alter food web dynamics, changing the quality and quantity of the food source at a critical time of development and seasonal growth, while diminished lipid stores upon meltout could result in fewer cells surviving dormancy. As lipids are the most energy dense of all biomolecules, taken together, such changes at the primary production level could result in less carbon transfer through the polar marine ecosystem.

### Supplementary Information

Supplementary Tables.

## Data Availability

All data are available in the open repository Figshare. 10.6084/m9.figshare.25222808.
