# Supplementary material for: Seasonal environmental transitions and metabolic plasticity in a sea-ice alga from an individual cell perspective
Source: Sci Rep. 2024 Jul 1;14:14984. doi: 10.1038/s41598-024-65273-0 (PMC11217269; doi:10.1038/s41598-024-65273-0)
Supplement: Supplementary file 1 — Supplementary Tables. [file 41598_2024_65273_MOESM1_ESM.docx]

**Supplementary Information:**

**Title: Seasonal transitions in environmental conditions and metabolic plasticity in a sea-ice alga: an individual cell perspective**

Authors: Rebecca J. Duncan, Janne E. Søreide, Daniel Nielsen, Øystein Varpe, Józef Wiktor, Mark Tobin, Vanessa Pitusi, Katherina Petrou

**Table S1:** Number of *Nitzschia frigida* cells measured per site, per date

| **Site** | **Date** | **Cells Measured** |
| --- | --- | --- |
| Inner Site | 6.4.22 | 12 |
|  | 21.4.22 | 34 |
|  | 29.4.22 | 30 |
|  | 13.5.22 | 30 |
| Outer Site | 7.4.22 | 9 |
|  | 20.4.22 | 30 |
|  | 30.4.22 | 33 |
|  | 12.5.22 | 30 |

**Table S2:** Statistical output of species-specific regression models. Multiple R^2^, adjusted R^2^, F statistic, associated degrees of freedom (DF) and p-value. Statistically significant p-values (< 0.05) are marked in bold.

|  |  | **Multiple R^2^** | **Adjusted R^2^** | **F Statistic** | **DF** | ***P* Value** |
| --- | --- | --- | --- | --- | --- | --- |
| **Outer Site** | Carbohydrates | 0.22 | 0.21 | 26.54 | 1,93 | **1.42 x 10^-6^** |
|  | Carboxylated Molecules | 0.60 | 0.58 | 135.9 | 1,93 | **2.2 x 10^-16^** |
|  | Lipid CH Stretch | 0.36 | 0.36 | 53.28 | 1,94 | **9.12 x 10^-11^** |
|  | Lipid (Ester Carbonyl) | 0.62 | 0.61 | 148.6 | 1,93 | **2.2 x 10^-16^** |
|  | Phosphodiesters | 0.38 | 0.37 | 55.86 | 1,92 | **4.12 x 10^-11^** |
|  | Protein (Amide II) | 0.39 | 0.39 | 60.16 | 1,94 | **1.04 x 10^-11^** |
|  | Saturated Fatty Acids | 0.57 | 0.56 | 122.6 | 1,94 | **2.2 x 10^-16^** |
|  | Silica | 0.60 | 0.59 | 135.7 | 1,93 | **2.2 x 10^-16^** |
|  | Unsaturated Fatty Acids | 0.52 | 0.52 | 102.5 | 1,94 | **2.2 x 10^-16^** |
| **Inner Site** | Carbohydrates | 0.14 | 0.13 | 15.61 | 1,100 | **1.45 x 10^-4^** |
|  | Carboxylated Molecules | 0.00 | 0.00 | 0.13 | 1,99 | 0.72 |
|  | Lipid CH Stretch | 0.26 | 0.25 | 34.89 | 1,100 | **4.87 x 10^-8^** |
|  | Lipid (Ester Carbonyl) | 0.21 | 0.21 | 26.99 | 1,99 | **1.10 x 10^-6^** |
|  | Phosphodiesters | 0.12 | 0.10 | 13.11 | 1,100 | **4.63 x 10^-4^** |
|  | Protein (Amide II) | 0.31 | 0.30 | 44.19 | 1,100 | **1.59 x 10^-9^** |
|  | Saturated Fatty Acids | 0.40 | 0.40 | 67.34 | 1,100 | **8.18 x 10^-13^** |
|  | Silica | 0.01 | 0.00 | 1.18 | 1,100 | 0.28 |
|  | Unsaturated Fatty Acids | 0.07 | 0.06 | 7.97 | 1,100 | **5.74 x 10^-3^** |
